# The Use of a Health Compliance Monitoring System During the COVID-19 Pandemic in Indonesia: Evaluation Study

**DOI:** 10.2196/40089

**Published:** 2022-11-22

**Authors:** Dewi Nur Aisyah, Logan Manikam, Thifal Kiasatina, Maryan Naman, Wiku Adisasmito, Zisis Kozlakidis

**Affiliations:** 1 Department of Epidemiology and Public Health Institute of Epidemiology and Health Care University College London London United Kingdom; 2 Indonesia One Health University Network Depok Indonesia; 3 Aceso Global Health Consultants Pte Limited Faculty of Public Health Universitas Indonesia Depok Indonesia; 4 Faculty of Public Health Universitas Indonesia Depok Indonesia; 5 International Agency for Research on Cancer World Health Organization Lyon France

**Keywords:** COVID-19, public health informatics, behavioral change, digital health, public health policy, monitoring, Asia, mask, social distance, mobile app, app, transmission, policy, health compliance

## Abstract

**Background:**

COVID-19 cases are soaring in Asia. Indonesia, Southeast Asia’s most populous country, is now ranked second in the number of cases and deaths in Asia, after India. The compliance toward mask wearing, social distancing, and hand washing needs to be monitored to assess public behavioral changes that can reduce transmission.

**Objective:**

This study aimed to evaluate this compliance in Indonesia between October 2020 and May 2021 and demonstrate the use of the *Bersatu Lawan COVID-19* (BLC) mobile app in monitoring this compliance.

**Methods:**

Data were collected in real time by the BLC app from reports submitted by personnel of military services, police officers, and behavioral change ambassadors. Subsequently, the data were analyzed automatically by the system managed by the Indonesia National Task Force for the Acceleration of COVID-19 Mitigation.

**Results:**

Between October 1, 2020, and May 2, 2021, the BLC app generated more than 165 million reports, with 469 million people monitored and 124,315,568 locations under observation in 514 districts/cities in 34 provinces in Indonesia. This paper grouped them into 4 colored zones, based on the degree of compliance, and analyzed variations among regions and locations.

**Conclusions:**

Compliance rates vary among the 34 provinces and among the districts and cities of those provinces. However, compliance to mask wearing seems slightly higher than social distancing. This finding suggests that policy makers need to promote higher compliance in other measures, including social distancing and hand washing, whose efficacies have been proven to break the chain of transmission when combined with masks wearing.

## Introduction

COVID-19, caused by the SARS-CoV-2 virus, remains a major global health threat. Since it was first identified in Wuhan, China, in December 2019, the virus has spread globally. As of May 2, 2021, the number of total confirmed cases stood at 151,812,556 cases, whereas the cumulative deaths reached 3,186,817. India ranks first in Asia (19,557,457 total cases and 215,542 deaths), whereas Indonesia ranks second in the region with 1,672,880 cases and 45,652 deaths as of May 1, 2021 [1].

Public health measures, in particular nonpharmaceutical interventions (NPIs), have been used consistently to reduce the likelihood of infections and community transmission [2,3]. Such measures include case isolation, voluntary home quarantine, social distancing, stopping mass gatherings, curfews, travels ban, lockdowns, as well as personal NPIs such as mask wearing, hand washing, and other health precautions. Mask wearing has been proven to be effective in reducing the likelihood of infections [4], yet its efficacy in reducing the risk of transmission is still being evaluated [4]. However, the evidence thus far indicates that when masks wearing is combined with regular hand washing and social distancing, they generally have a positive impact toward reducing SARS-CoV-2 transmissions [4]. Community mask wearing can prevent infected persons and protect uninfected wearers, which reduced the risk of infection by 79% [5-7]. In accordance with policies for disciplinary enforcement, mandating face mask use in public have been associated with a significant decline in reducing infection rates in 15 US states when comparing before and after mask mandates [8]. Furthermore, the timing in implementing such NPIs in relation to the curve of the epidemic and the population’s adaptation through behavioral changes are main factors contributing to the success of NPIs [9,10].

Within this context of pandemic control, establishing the basic reproduction number of COVID-19 is critical in predicting herd immunity targets and having a relative measure of effectiveness for public health interventions [11]. However, another critical element beyond the reproduction number is the need for rapid and widespread behavioral change that remains adaptable to the changing conditions [12]. Behavioral change allowing the implementation of the NPIs mentioned above needs to be articulated clearly and internalized collectively [13], in conjunction with socioeconomic activities that aim to allow society to remain productive and safe as a whole [14].

During the current pandemic, there are some examples where existing capacities were activated and enhanced coordination mechanisms across multiple sectors, as well as toward establishing monitoring evaluation systems, thus introducing large-scale behavioral change by using health technology [15]. For instance, South Korea has invested from the outset of the pandemic in digital health solutions as a means of strengthening surveillance capacity, aided by the use of a national smartphone app for tracing and tracking infected people by GPS and combining this information with other public health measures [16-19]. Therefore, health technology applications have started to emerge as potential key solutions in the control of the COVID-19 pandemic, beyond the tracing aspect and often including advice or recommendations on personal preventive aspects, resulting to a behavioral change that could be also monitored in terms of its health protocol compliance [17,20-22].

Indonesia is administratively divided into 34 provinces and 514 cities and regencies, with independent local governments and parliamentary bodies. Often, health policies are decided at the federal level and implemented at the provincial level, as in the case of infectious disease surveillance for zoonotic diseases. The nationwide health care infrastructure includes 10,138 public health centers (*Pusat Kesehatan Masyarakat*; primary health care facilities) and 2902 hospitals (tertiary health care facilities) spread across these provinces, of which 132 hospitals are designated as national referral centers for the treatment of COVID-19 [23]. As such, the centralization of the COVID-19 response represented a departure from the routine implementation of health care response policies.

The government of Indonesia—the world’s fourth most populous nation—has taken NPIs to promote behavioral changes, collectively termed as “health protocol.” The health protocol consists of mask wearing (*Menggunakan masker*), hand washing (*Mencuci tangan*), and social distancing (*Menjaga jarak*). The government promoted it consistently under the popularized “3M” acronym (from the initial of each action in the Bahasa Indonesian language) and monitored public compliance thereof. This study aimed to evaluate compliance to the health protocol in public spaces between October 2020 and March 2021, thus including the entire second wave of the pandemic in Indonesia. Importantly, the data used here have been collected from the Indonesia National Task Force for the Acceleration of COVID-19 Mitigation by using the *Bersatu Lawan COVID-19* (BLC) digital monitoring app. This represents the first time in which an app for digital health, introduced nationally, produced data able to be analyzed on a real-time basis and using an integrated approach. Importantly, the system uses observer-reported compliance, thus this app is able to minimize bias from self-reported data. Additionally, this paper will describe and discuss how such data allowed the Indonesian government agencies to monitor health protocol compliance among the Indonesian public and in turn inform policy making.

## Methods

### The BLC Integrated System

The BLC is an integrated information system built by the National Task Force for the Acceleration of COVID-19 Mitigation. The task force was formed by the President of the Republic of Indonesia to perform, control, monitor, create, and implement strategic policies to accelerate national COVID-19 responses [24]. In performing those duties, it needed, created, and used an enhanced data reporting system to bring together and produce an in-depth analysis of the available COVID-19 information. This system aims to describe case distribution and determine the zoning of the COVID-19 transmission level, including health protocol compliance monitoring. It is first system of this kind in the country that used a big data approach, with real-time, systematic, and interoperable processes for delivering evidence-based policies [25]. The BLC system integrates data from many sectors. For example, it contains health care data (laboratory, hospital, and surveillance data) from the Ministry of Health; public transportation data; educational data from the Ministry of Education and Culture; logistics data regarding the vaccination rollout, etc. These data are obtained through the connection of different databases at ministries and agencies and are made accessible through a single interface.

### The Health Protocol Compliance Monitoring System Using BLC

The BLC health protocol compliance app was developed from May to July 2020. Initially, it was designed as a means of helping Indonesian frontline public order forces (such as police and military) to move from paper-based to digital reports in monitoring compliance to the newly implemented public health restrictions (September 2020). In comparison to paper-based reports, digital reports are easier to compile and analyze. Consequently, app use included monitoring the compliance during potentially high-risk national events, such public holidays and regional elections or election campaigns. The subsequent step of app use extension (October 2020) was the inclusion of volunteers from the general public, termed behavioral change ambassadors (*Duta Perubahan Perilaku*). Behavioral change ambassador are individuals who volunteered for this role and are from a wealth of backgrounds and age groups—for instance, including from students to university lecturers, Civil Service Police Unit (*Satpol PP*) personnel, as well as from many other sectors. They are required to have digital literacy so that app use is as complete and accurate as possible and can report the data daily during their activities, particularly in monitoring the wider public health protocol adherence.

As of May 2, 2021, the app had 437,093 registered users, of which 97,598 were military personnel, 253,984 were police services personnel, and 85,511 were members of the public/ambassadors. The app itself contains a training module, showing users how to generate an account for personnel in the field, how to report data, and how to understand the dashboard’s statistic results. As the monitoring can be an entry from all Indonesian levels, the account given is generated based on the regional levelling access.

**Figure 1:**
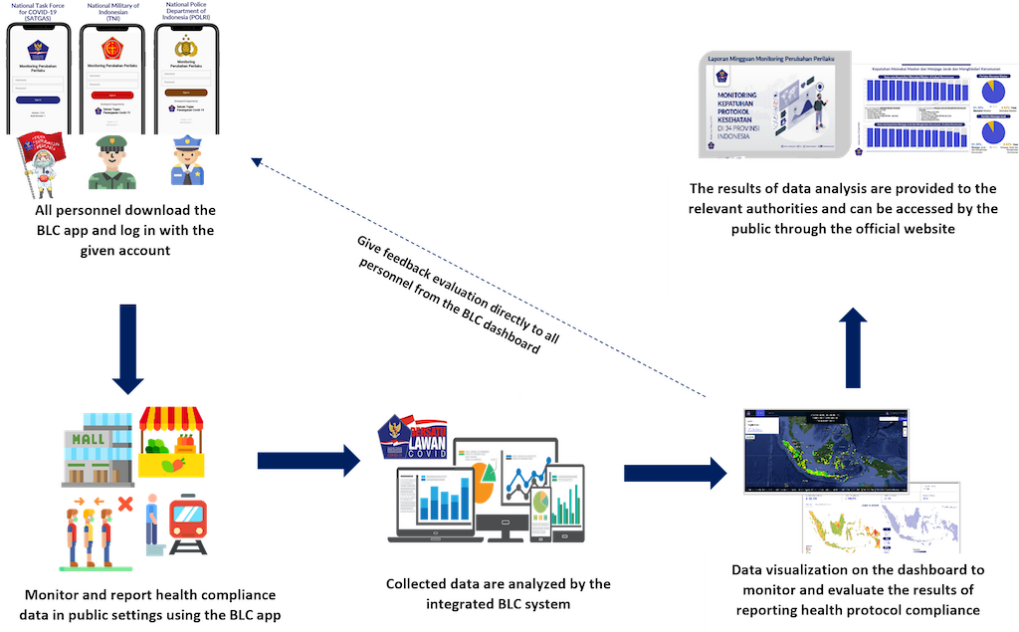
Overview of health protocol compliance monitoring at public places. BLC: Bersatu Lawan COVID-19. Higher-resolution version of this figure is available in Multimedia Appendix 1.

The monitoring system was reported in real time using the BLC behavioral change app at public places, which tend to be crowded locations, such as markets, recreational areas, shopping malls, restaurants, places of worship, offices, train stations, bus terminals, airports, sport centers, schools, etc. Those locations were chosen based on the tendency or potential for crowds to become a place for clusters of COVID-19 transmission. Several studies have found the potential for transmission both indoors and outdoors, such as in transit places, restaurants, fitness centers, places of worship, schools, supermarkets, etc [26,27]. The reports sent include a photo of the monitoring results and an input data questionnaire by all personnel in the field. When the report data have been received, the integrated BLC system will analyze them into statistical data to determine location mapping to improve health protocol compliance. Furthermore, the information based on report data will be visualized and monitored through the BLC integrated dashboard accessible to all levels (central government, provinces, cities, districts, and subdistricts; Figure 1).

The reporting personnel from the military (*Tentara Nasional Indonesia*) and police force (*Kepolisian Negara Republik Indonesia*) are given incentives by their respective agencies, whereas the behavioral change ambassadors are community volunteers who do not receive incentives. All of them will continuously report any potential crowds and compliance levels in the local community wherever they are. In addition, there is no limit to the number of reports a person can submit per day. The emphasis is on generating objective reporting that shows compliance conditions in the field and inputting data correctly.

To ensure validation and have quality control measures, first, all personnel were provided with training on how to complete the report, and second, the report can be populated only using specific parameters and within a specific range for each variable. The report also contains mandatory fields for the collected variable; otherwise, it cannot be submitted within the system. In this way, we allow for a standardized, common, and minimum data set of information to be collected across all locations, hence allowing the real-time creation of the dashboards.

Furthermore, the quality control is conducted by having a regular randomized check by an operator at upper levels of the system (for example, reports at district levels are monitored at the provincial level). This routine monitoring process considers the number of reports collected per area, the reporting locations, the number of personnel submitting reports, as well as the quality of reports submitted. The latter is checked manually, that is, counting how many individuals in a given photo are wearing masks. If the report contains erroneous information, the person who submitted the report will be given a warning message. If a second report by the same person fails the quality control, then further reports by this person might be disqualified.

Additionally, if the personnel do not submit a complete report in the app, a warning will appear to urge the user to complete the data input. If duplications are found, the system will automatically delete data from the same villages or subdistricts (*kelurahan*).

### Data Collection

**Figure 2:**
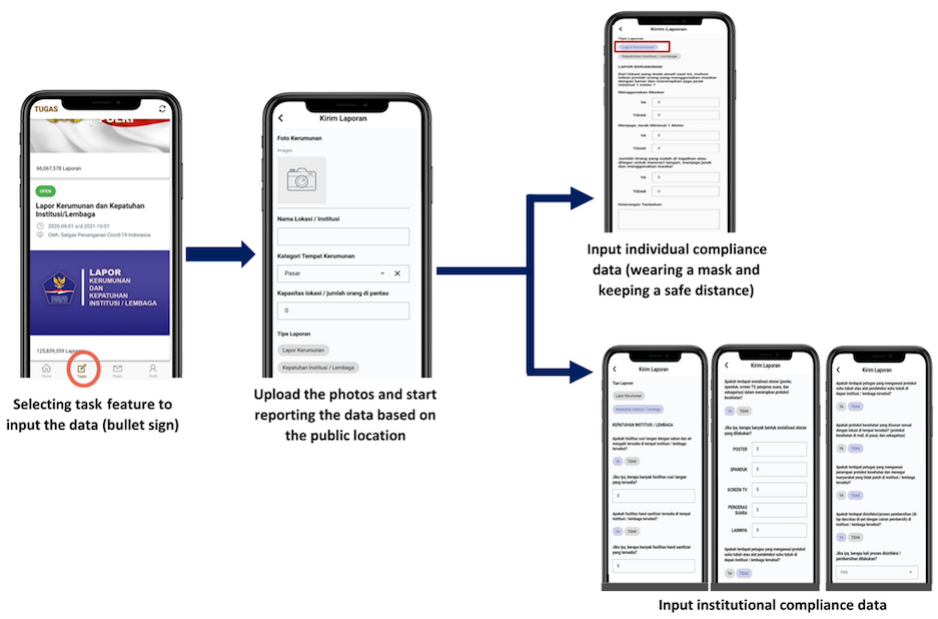
Data input using the BLC behavioral change app. BLC: Bersatu Lawan COVID-19.

Data input is started by selecting the task feature in the app. There are 2 levels of compliance that are monitored, namely individual compliance and institutional compliance. Individual compliance consists of compliance with mask wearing and social distancing as well as avoiding crowds. Institutional compliance consists of monitoring hand washing facility availability, socialization of the application of health protocols, body temperature checks (using a thermo-gun or thermal body), the presence of health protocol supervisory officers, and regular disinfection activities. All personnel may frequently input data to report health protocol compliance and monitor crowd activities around them based on reporting the location using GPS (Figure 2). Some personnel can submit live report by taking photos in the field, whereas others can submit several delayed reports by the end of each day through saved pictures from their photo gallery. The National COVID Task Force actually recommends that the personnel send live reports. However, not all personnel are equipped with stable internet connection and reliable cellular phone all day long; thus, delayed reporting should still be allowed for their convenience.

### Data Analysis

The BLC system will automatically analyze the data collected to generate results and visualize the information on simplified output graphs, collectively presented on a dashboard. The data can be queried using a set of predetermined variables, based on big data analytics infrastructure. For instance, the proportion of masked individuals are calculated by dividing the number of people wearing masks by the sum of people present within a given locality (as provided by the photographic evidence). Likewise, the proportion of social distancing compliance is obtained by dividing the number of people keeping social distance by the number of people present at a given locality (the number of people who keeping social distance is calculated by using the photographic evidence).

The result also is analyzed for regional compliance zoning, both in individual and institutional compliance. These are based on the personnel’s observed report on individual and institutional compliance. The map is then zoned for compliance to mask wearing and social distancing, and the data are updated in real time to show how many cities/districts have compliance levels of <60% (red), 61%-75% (orange), 76%-90% (yellow), and 91%-100% (green).

Additionally, the BLC systems also presents “institutional compliance,” which refers to places or locations where crowds are likely to converge, namely markets, recreational areas, shopping malls, restaurants, places of worship, offices, train stations, bus terminals, airports, sport centers, schools, etc. Their compliance is then divided into 4 categories: “noncompliant” institutions (0%-35% compliance rate), “less compliant” institutions (35.01%-65% compliance rate), “compliant” institutions (65.01%-85% compliance rate), and “very compliant” institutions (85.01%-100% compliance rate).

### Taxonomy of the Health Protocol Compliance Monitoring System

According to the typology of digital public health tools from Gasser et al [28], the typology is based on 4 main categorial variables—that is, key actors, data types, data source, and model of consent. In this system, based on the typology, our key actors are government and citizens. The data types for this system are categorized as nonsensitive, whereas the data source come from IP, GPS, and citizens. The consent is categorized as opt-in. These typologies can also identify 4 main functional categories of digital public health technologies for pandemic management, such as proximity and contact tracing, symptom monitoring, quarantine control, and flow modeling. The system for monitoring compliance presented here is the closest aligned (although not entirely overlapping) with the quarantine compliance functional category [28].

Furthermore, based on the Behavior Change Techniques taxonomy from Michie et al [29], this app was categorized in the group “Feedback and monitoring,” particularly within subgroup 2.1 “Monitoring of behavior by others without feedback.” Observing people in crowd locations for mask wearing and social distancing is part of data collection, with the person’s knowledge being part of the behavior change strategy to reduce the risk of COVID-19 transmission [29].

In terms of ethics, especially protecting privacy, the health protocol compliance monitoring system does not collect individual-level data. The data are collected in an aggregated format. The only individual data embedded in the system are the personnel identities of the app operators, which they have to provide so that they can complete the information input. However, this information is limited only within their own respective institutions (eg, armed or police forces) and not open to the public. In terms of preserving autonomy, the use of the compliance monitoring app is not compulsory but based on the voluntary commitment of the data providers. The app does not contain data that could be used for discrimination (eg, race, ethnic group, gender, etc); however, some areas of the country could be identified as better or worse performing at the population compliance level. This is unlikely to generate discrimination as defined by Gasser et al [28], although it might result in additional temporary restrictive measures. Finally, there are active, ongoing discussions as to a potential expiration for the collected data. However, no decision has been reached yet.

In addition, we also considered the reactivity of the subject/community during system design and development, although it was not considered an issue. The BLC app monitoring system was developed to answer the data needs related to the compliance of the Indonesian people. Indonesian frontline public order forces (such as military and police) were chosen as observers because they have the main function/duties in enforcing discipline and have already been trained in dealing with the public at large while respecting legal and ethical norms. The National police department of Indonesia (*Kepolisian Negara Republik Indonesia*) has the authority to issue warnings, fines, and social sanctions. This is in accordance with the instructions of the president and the commanders of the National Military and the National Police Chief [30].

### Ethics Consideration

We declare that the data collected for this paper do not require ethical approval, because they are made available to the public by the National Task Force for the Acceleration of COVID-19 Mitigation on their website [31].

## Results

### Real-time Health Protocol Compliance Monitoring Report

The total number of reports gathered through BLC between October 1, 2020, and May 2, 2021, was more than 165 million, with 469 million people’s behaviors monitored, observed in 124,315,568 locations in 514 districts/cities across 34 provinces in Indonesia (ie, near complete national coverage, as also explained below). Additionally, within the same period, over 508,000 institutions were observed in more than 41,235,847 locations in 504 districts/cities.

This system always received more than 680,000 reports per 24 hours as of May 2, 2021, the end of this observation period. This system also received over 2500 reports per minute and reached a peak capacity of 1894 reports per second on April 14, 2021.

**Figure 3:**
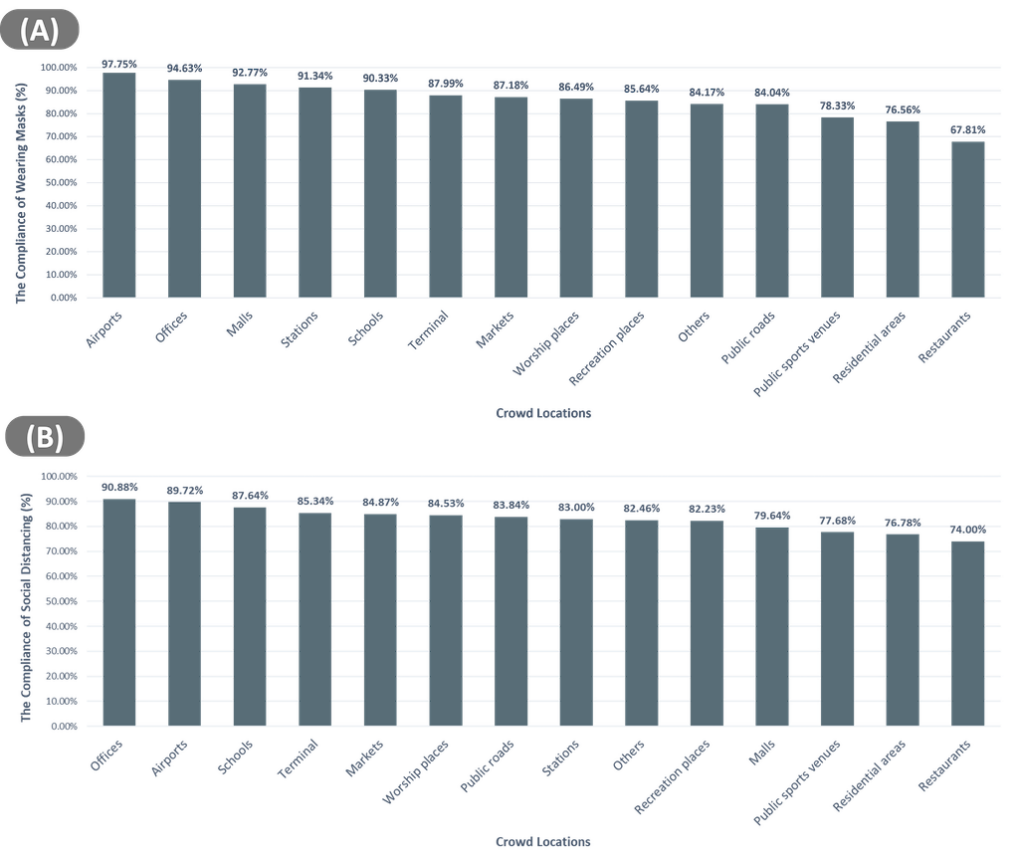
The average of the compliance of (A) wearing marks and (B) social distancing at crowded locations cumulatively (reports submitted from October, 1 2020, to May 2, 2021). Locations are divided by function and identified as areas of the highest risk for COVID-19 transmission.

The overall national figures received through BLC showed 85.89% (322,736,010/375,711,304) of the observed individuals wearing masks and 14.11% (52,975294/375,711,304) not wearing masks. Similarly, 84.13% (315,973,207/375,711,304) of people kept social distancing and 15.8% (59,738,097/375,711,304) did not, as a cumulative estimate. Figure 3 shows locations ranked according to mask-wearing and social distancing compliance.

**Figure 4:**
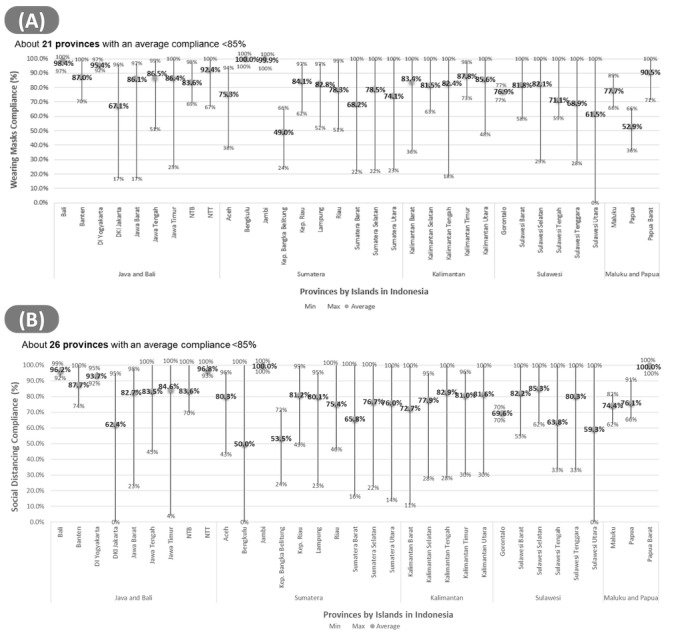
The number of the lowest, highest, and average compliance rates for (A) wearing masks and (B) social distancing from all districts/cities in 34 provinces, calculated in the last 7 days as of May 2, 2021. (There were no reports for the last 7 days in North Maluku Province). DI: Daerah Istimewa; DKI: Daerah Khusus Ibukota; Kep.: Kepulauan; NTB: Nusa Tenggara Barat; NTT: Nusa Tenggara Timur.

Figure 4 demonstrates how the same information can become more granular, incorporating the relative proportion of compliant/less-compliant categories to the cumulative total. The line in the middle of graphs within Figure 4 shows the range; the longer the line in the box plot, the greater the variation in the data.

Figure 4 presents this information according to the provinces in Indonesia. It is estimated that 11 provinces have average compliance rates more than 85% (Bali, Daerah Istimewa Yogyakarta, Daerah Khusus Ibukota Jakarta, East Java, Riau Island, Central Kalimantan, East Kalimantan, North Kalimantan, West Sulawesi, North Sulawesi, and West Papua), and this rate is lower for all other provinces.

**Figure 5:**
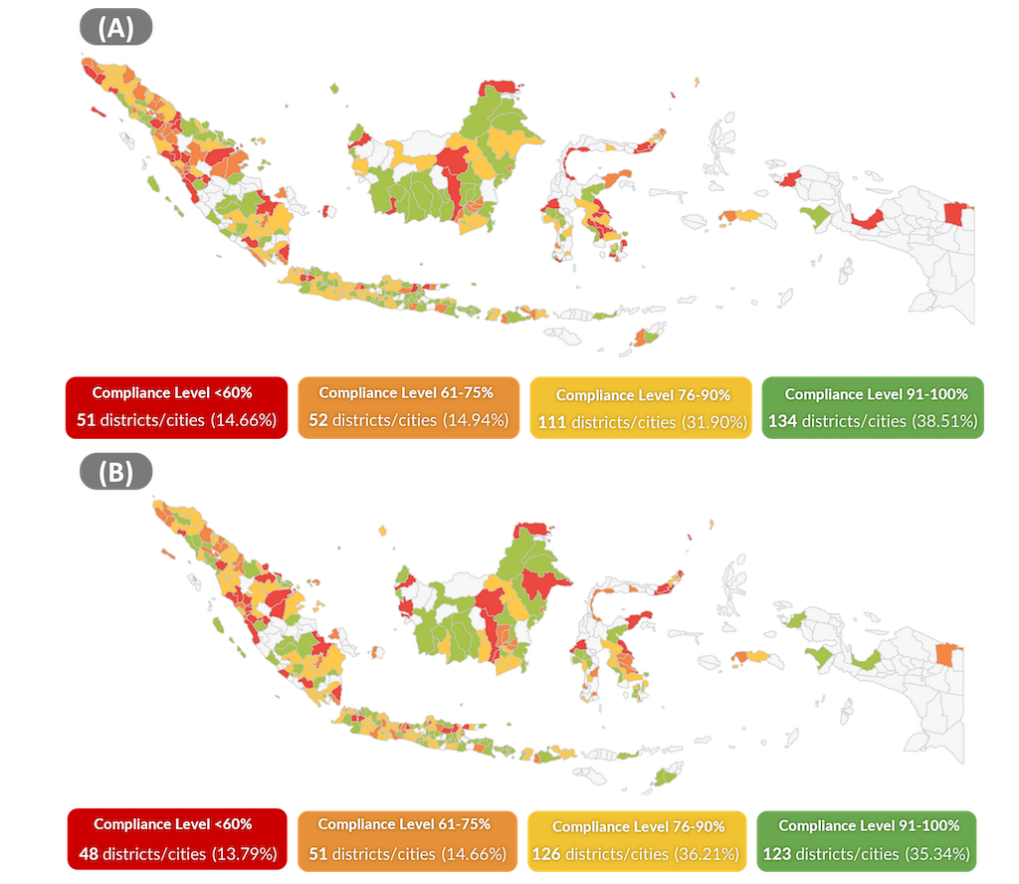
The zoning map of (A) wearing masks and (B) social distancing compliance, calculated in the last 7 days as of May 2, 2021.

Figures 5 maps this provincial variation. Out of a total of 348 districts/cities visualized, Figure 5 shows the estimated mask-wearing compliance rate with 51 (14.66%) districts/cities in the red zone, 52 (14.94%) in the orange zone, 111 (31.9%) in the yellow zone, and 134 (38.51%) in the green zone. For social distancing compliance, Figure 5 shows that 48 (13.79%) districts/cities were in the red zone, 51 (14.66%) were in the orange zone; 126 (36.21%) were in the yellow zone; and 123 (35.34%) were in the green zone.

**Figure 6:**
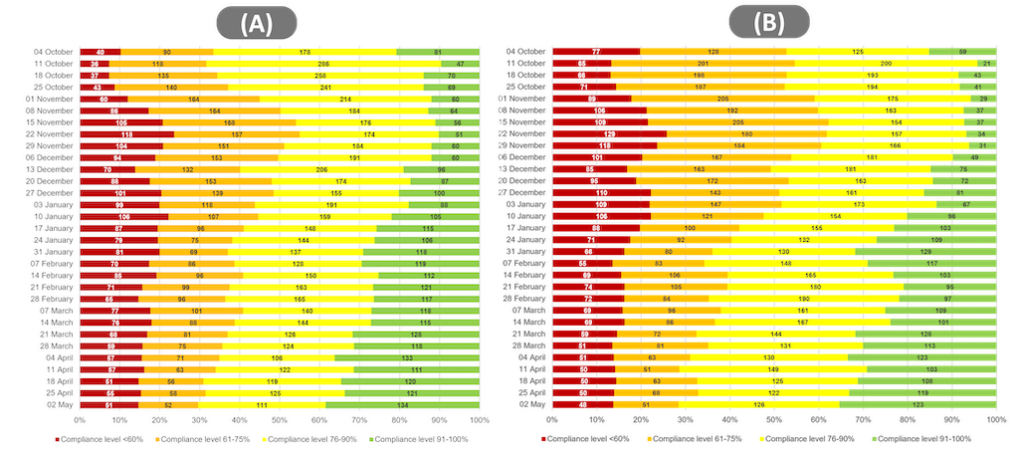
The compliance zoning development by the number of districts/cities in a weekly period from October 4, 2020 to May 2, 2021: (A) mask wearing and (B) social distancing.

In terms of a wider view—and one that can be linked to NPI announcements—Figure 6 shows a weekly average of the cumulative compliance rates for the 2 categories mentioned above. Overall, estimated compliance fell in November and December (before the peak of the second wave of the pandemic), whereas it increased from January to May 2021 (during and after the second wave).

**Figure 7:**
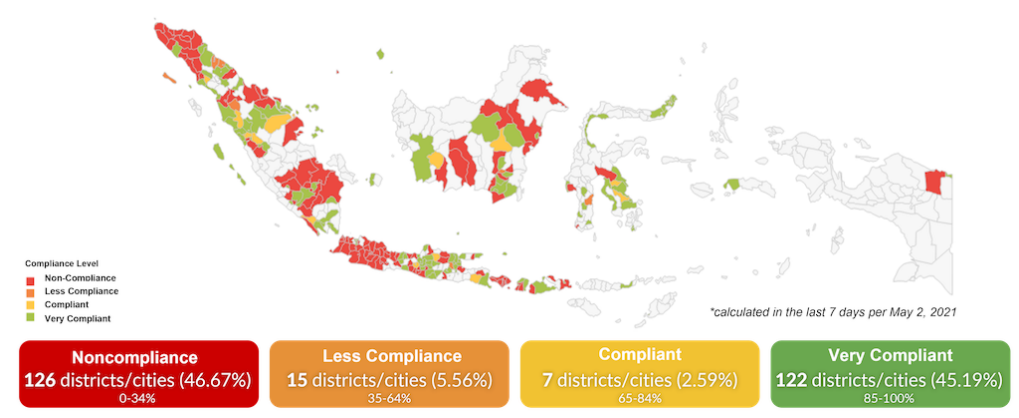
The zoning map of institution compliance, calculated in the last 7 days leading up to May 2, 2021.

**Figure 8:**
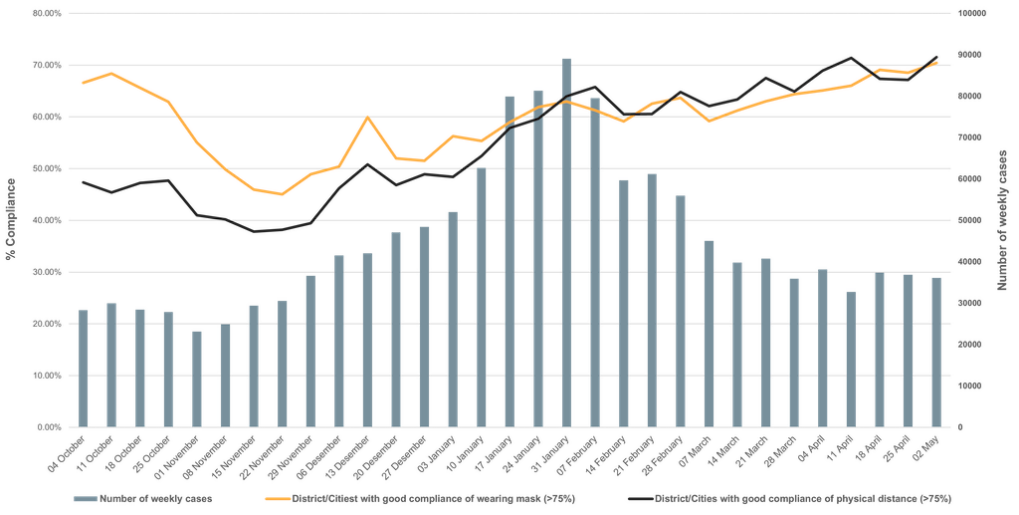
Proportion of health protocol compliance in relation to the weekly number of COVID-19 cases.

Figure 7 shows institutional compliance across Indonesian districts and cities: 126 (46.67%) districts had a high rate of noncompliant institutions, 15 (5.56%) had a smaller rate of noncompliant institutions, 7 (2.59%) had compliant institutions, and 122 (45.19%) had very compliant institutions. Figure 8 shows the association of health protocol compliance in relation to the weekly number of COVID-19 cases during the second wave in 2020 (this is the first wave where the mobile app was implemented). The graph shows that there was a lower level of compliance before the advent of the second wave and that compliance rose significantly as the wave progressed and as further public health measures were introduced and monitored.

## Discussion

### Principal Findings

Despite variation between provinces and among districts and cities, which are illustrated using 4 colored zones, the overall majority of districts/cities demonstrate medium to high compliance for both mask wearing and social distancing. Compliance to mask wearing is higher than to social distancing, as would be expected for being an easier element to be controlled at an individual level. These results demonstrate good correspondence to the survey results from the Indonesian National Bureau of Statistics, conducted in September 2020, which showed 91.98% of respondents always wearing masks when leaving their homes, as opposed to 73.53% of respondents who stated that they always keep social distancing when leaving their homes [32]. Furthermore, the Indonesian National Bureau of Statistics results showed that more than half of the respondents self-stating their own noncompliance thought there were no penalties from the authorities for such noncompliance; more than a third did not comply as they could not see or hear a COVID-19 case in their immediate familial environment; and nearly all noncompliant individuals perceived the protocols as disturbing for them in performing their jobs [32].

This seemingly higher compliance with mask wearing compared to social distancing is an interesting finding despite the complication and hesitancy of mask wearing observed across the globe [33] and the limited evidence available during the time of the reported observations to claim its efficacy in breaking the chain of transmission [4,19]. Seeing the relatively high reported compliance that countries, such as Indonesia, not used to wearing masks routinely are able to do so is a positive sign for the penetration of the public health messages. However, compliance is variable between different types of activities, and as such, the messaging might have to be nuanced to promote other measures, such as hand washing, that are the most effective at a population level when combined with masks wearing [7,34].

The results have shown the variation of compliance rate between provinces and among the districts and cities in provinces. However, Bali and Daerah Istimewa Yogyakarta provinces have average compliance rates of more than 85% in mask wearing and social distancing. This finding might be due to the high number of field personnel from the police and military who are deployed in these provinces to ensure health protocol compliance [35,36]. These provinces are among the most popular tourist destinations in Indonesia [37], with a higher likelihood of crowd gathering and thus attracting a higher level of policing.

For future references, the insights on health protocol compliance monitoring across all 34 provinces are updated regularly on the National Task Force for the Acceleration of COVID-19 Mitigation website [31], with the latest one posted on September 25, 2022 [38].

### Strengths and Limitations

Therefore, the number of reports generated by this BLC behavioral change app might be restricted by the numbers and locations of reporters. Nevertheless, this study has revealed the insights from a digital reporting system that can benefit policy makers in monitoring behavioral changes when the reporting is done comprehensively and using big data analytics. One of the factors supporting this monitoring’s success is its real-time data collection at a micro-scale, based on cloud technology. This enables data interconnection among districts, cities, and provinces, which can be analyzed altogether by the Indonesia National Task Force for the Acceleration of COVID-19 Mitigation. In a large and decentralized country such as Indonesia, data interconnection is key to obtaining national analysis and informing effective evidence-based policies.

Police and military forces have made major contributions to supplying these real-time data. Although military forces involvement in a health crisis remains a contested idea [39], this case can be an additional example of the essential roles of the police and military in COVID-19 response within Indonesia’s large territory [40].

However, this study also has certain limitations. First, the app is provided only for users who have Android smartphones. Second, human errors are still found in the reports, such as irrelevant pictures being uploaded to the system. Third, the reporters are limited to personnel and ambassadors in several public spaces. In the future, this app might expand the reporters to the wider public to generate reports from more categories of public spaces.

### Conclusion

To conclude, this paper has demonstrated the importance of promoting NPIs to prevent COVID-19 transmission and case surge. These interventions require public behavioral changes to wear masks, keep social distancing, and wash hands frequently. This paper discovers that the need to monitor these behavioral changes can be done through a mobile app. Therefore, this paper discusses the example of the BLC behavioral change app as used in Indonesia, the most populous country in Southeast Asia, whose COVID-19 cases are ranked second in Asia, after India, to date.

This paper discusses the multisectoral coordination behind the development and report submissions to this app, which includes police officers, military personnel, and community ambassadors. It further discovers how the big data analytics have been used to analyze these reports on a weekly basis to provide updates to policy makers and inform government COVID-19 response policies through the Indonesia National Task Force for the Acceleration of COVID-19 Mitigation.

Based on the data gathered through the app during the period from October 1, 2020, to May 2, 2021, it is apparent that compliance rate varies among the 34 provinces and among the districts and cities of those provinces. However, it is interesting to find that compliance to mask wearing seems to be slightly higher than social distancing. Although this can be a positive finding on behavioral change promotion, policy makers need to promote higher compliance in other measures, including social distancing and hand washing, whose efficacies have been proven to break the chain of transmission when combined with mask wearing. Nevertheless, this app has provided data that can inform public behavior patterns, which can inform policy makers to take the necessary actions to prevent a surge in COVID-19 cases.

## Appendix

Multimedia Appendix 1 Higher resolution version of Figure 1. Overview of health protocol compliance monitoring at public places. BLC: Bersatu Lawan COVID-19.

## Abbreviations

BLC: Bersatu Lawan COVID-19
NPI: nonpharmaceutical intervention
