# Supplementary figures and images for: The Use of a Health Compliance Monitoring System During the COVID-19 Pandemic in Indonesia: Evaluation Study
Source: JMIR Public Health Surveill. 2022 Nov 22;8(11):e40089. doi: 10.2196/40089 (PMC9683531; doi:10.2196/40089)

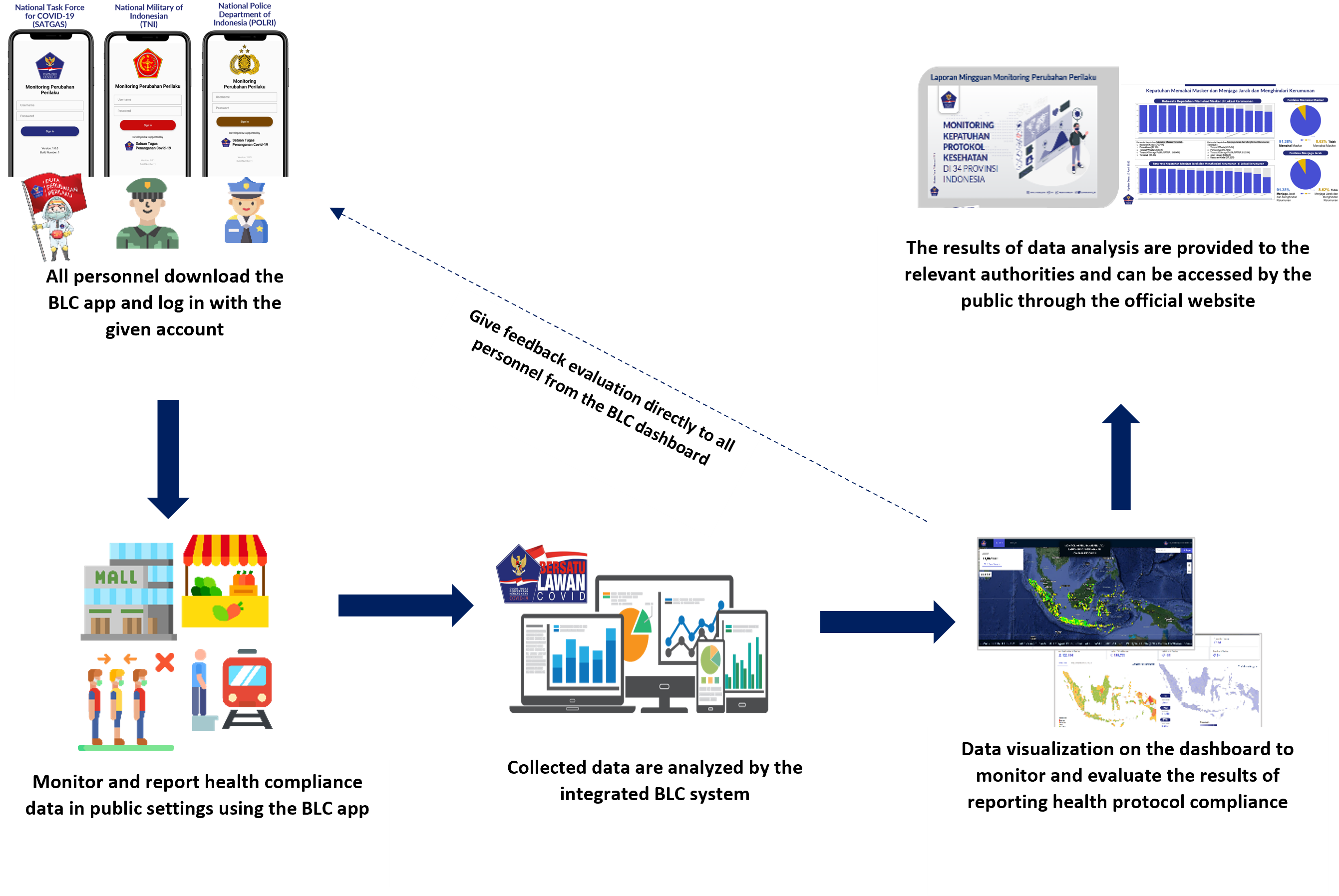

Supplement: Multimedia Appendix 1 [file publichealth_v8i11e40089_fig.png]
